# Low-cost, open-source *XYZ* nanopositioner for high-precision analytical applications

**DOI:** 10.1016/j.ohx.2022.e00317

**Published:** 2022-05-19

**Authors:** Hsien-Shun Liao, Christian Werner, Roman Slipets, Peter Emil Larsen, Ing-Shouh Hwang, Tien-Jen Chang, Hans Ulrich Danzebrink, Kuang-Yuh Huang, En-Te Hwu

**Affiliations:** a Department of Mechanical Engineering, National Taiwan University, Taipei 10617, Taiwan; b Physikalisch-Technische Bundesanstalt, Bundesallee 100, Braunschweig 38116, Germany; c The Danish National Research Foundation and Villum Foundation’s Center for Intelligent Drug Delivery and Sensing Using Microcontainers and Nanomechanics (IDUN), Department of Health Technology, Technical University of Denmark, Kgs. Lyngby 2800, Denmark; d Institute of Physics, Academia Sinica, Taipei 11529, Taiwan

**Keywords:** Nanopositioning, 3D printing, Atomic resolution, Vibrometer, Atomic force microscopy, Scanning electron microscopy, IDUN, Intelligent Drug Delivery and Sensing Using Microcontainers and Nanomechanics, PZT, Piezoelectric actuator, DAC, Digital-to-analog converter, OSF, Open Science Framework, PLA, Polylactic acid, OPU, Optical pick-up unit, AFM, Atomic force microscope, SEM, Scanning electron microscope, DVD, Digital video disc, HOPG, Highly oriented pyrolytic graphite

## Abstract

Nanoscale positioning has numerous applications in both academia and industry. A growing number of applications require devices with long working distances and nanoscale resolutions. Friction–inertia piezoelectric positioners, which are based on the stick–slip mechanism, achieve both nanometer resolution and centimeter-scale travel. However, the requirements of complex preload mechanism, precision machining, and precise assembly increase the cost of conventional friction–inertia nanopositioners. Herein we present the design of an open-source *XYZ*-axis nanopositioning system. Utilizing a magnet-based stick–slip driving mechanism, the proposed *XYZ* nanopositioner provides several advantages, including sub-nanometer resolution, a payload capacity of up to 12 kg (horizontal), compact size, low cost, and easy assembly; furthermore, the system is adjustment-free. The performance tests validate the precision of the system in both scanning and stepping operation modes. Moreover, the resonant spectra affirm the rigidity and dynamic response of the mechanism. In addition, we demonstrate the practical applications of this nanopositioner in various measurement techniques, including scanning electron microscopy, vibrometry, and atomic force microscopy. Furthermore, we present 11 variations of the nanopositioner designs that are either compatible with ultra-high-vacuum systems and other existing systems, 3D printable, or hacking commercial linear slides.

## Graphical abstract

**Figure:**
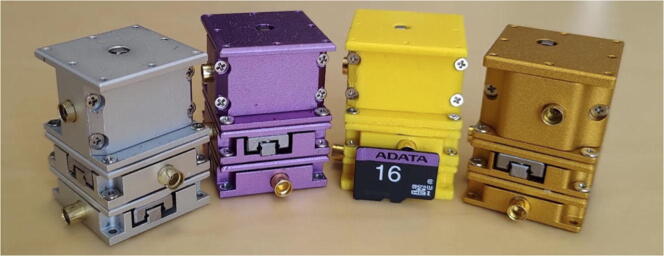

## Hardware in context

Precision positioning, which is key to nanoscale control, measurements, and manufacturing, has wide-ranging applications in both academia and industry [1], [2], [3], including nanotechnology [4], [5], [6], nanometrology [7], [8], [9], bioengineering [10], semiconductor fabrication [11], and aerospace [12]. Piezoelectric materials have numerous advantages, such as sub-picometer resolution, long service lifetimes, and compact sizes. Therefore, various types of piezoelectric positioners have been developed to achieve sub-nanometer precision [13], [14], [15]. For instance, flexure-guided piezoelectric positioners are widely used in microscopic imaging, nanoscale manipulation, and nano-machining [16], [17], [18]. However, the maximum displacement of the flexure-guided mechanism is usually less than a few hundred micrometers [19].

Friction–inertia piezoelectric nanopositioning systems utilize a stick–slip mechanism that can achieve both high resolutions and long traveling ranges [20], [21], [22], [23], [24]. In this mechanism, a sawtooth waveform drives the piezoelectric actuator, generating alternate slow and rapid motion. During the slow-moving period, the piezoelectric actuator moves a slider through a friction force. In the rapidly moving period, the inertia of the slider holds it in position and completes the stepping cycle. This mechanism requires a precisely adjusted preload force on the slider to maintain the friction force in the appropriate stick–slip zone [25]. The preload is often provided by a flexure structure, which requires precise electric discharge machining [26], [27], [28]. The requirements of a complex preload mechanism and precision machining result in an increase in the cost of nanopositioning systems.

**Fig. 1:**
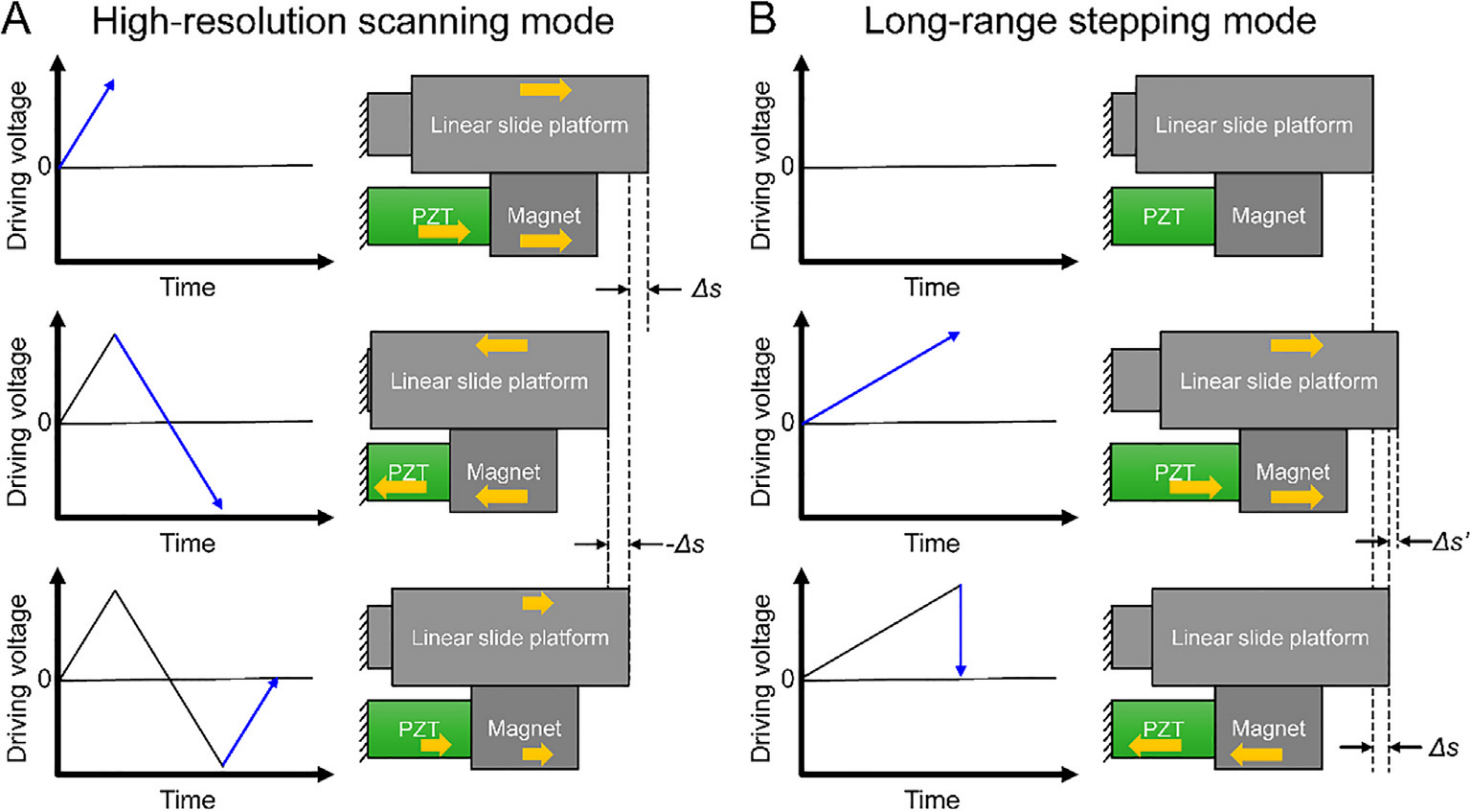
Operating modes of the magnet-based driving mechanism. A magnet provides friction force, whereas a piezoelectric actuator (PZT) drives a linear slide platform. **A)** High-resolution scanning mode. Positive and negative voltage ramps respectively drive the PZT for *Δs* and *-Δs* distance actuation. **B)** Long-range stepping mode. A sawtooth waveform drives the PZT and achieves *Δs* displacement with a *Δs’* backlash.

Herein, we describe a simple, adjustment-free, and open-source *XYZ* nanopositioner that has a compact size, low cost, easy assembly, and high-vacuum compatibility. The proposed open-source nanopositioner utilizes a magnet-based driving mechanism; the magnet simultaneously provides both a preload force and a friction surface. Through the stick–slip driving mechanism, the open-source nanopositioner can perform both high-resolution scanning (Fig. 1A) and long-range stepping (Fig. 1B) by the application of triangular and sawtooth waveforms, respectively. Moreover, the magnet significantly simplifies the driving mechanism and avoids fatigue issues that plague flexure structures.

## Hardware description

**Fig. 2:**
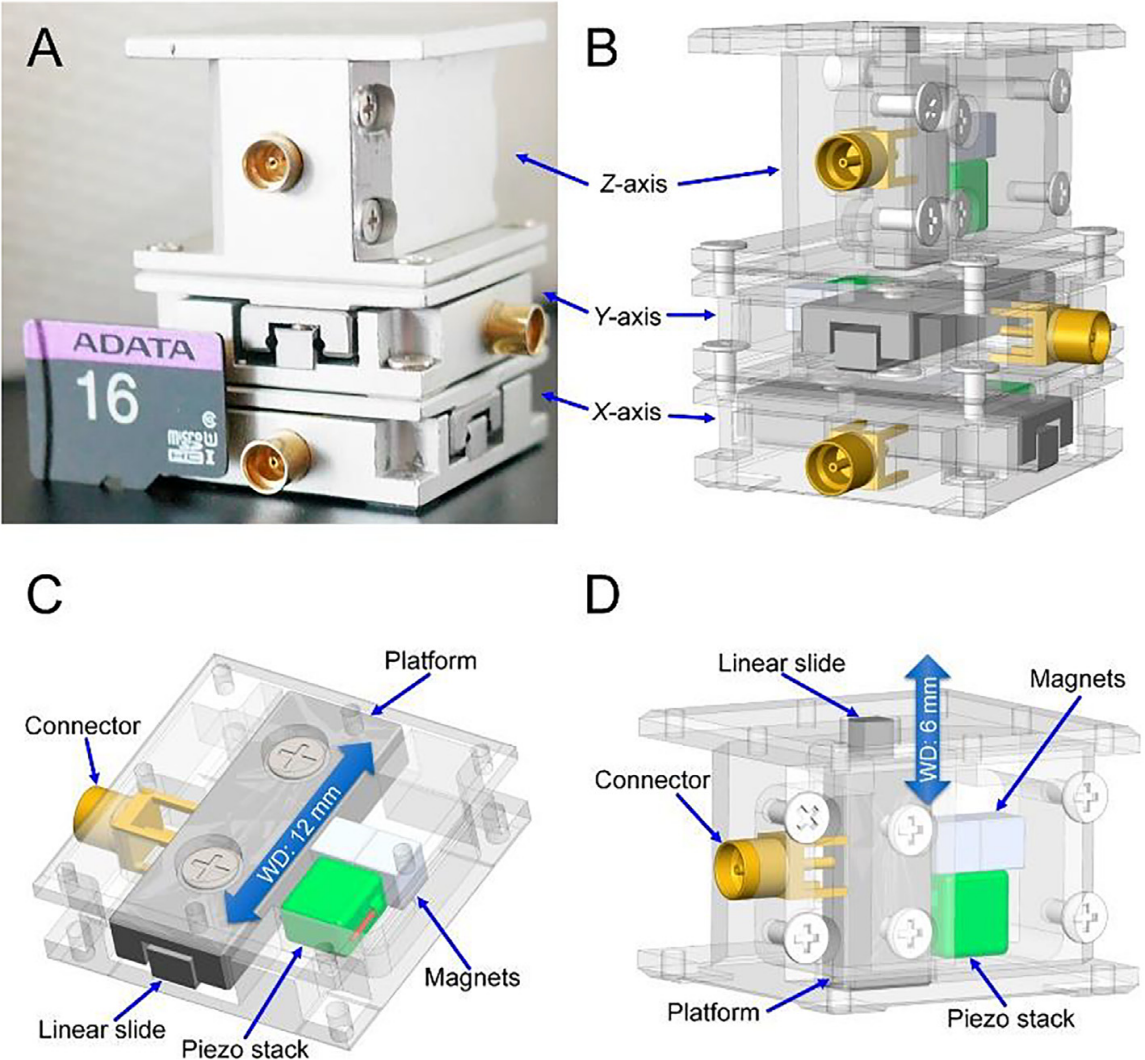
Overview of the open-source *XYZ* nanopositioner. **A)** Photo of the *XYZ* nanopositioner. A micro-SD card (11 mm × 15 mm), shown for comparison, demonstrates the compact size of the positioner. **B)** Computer-aided design drawing of the *XYZ* nanopositioner. **C)** Magnetic driving mechanism design of the *X* (horizontal)-axis linear nanopositioner with a working distance of 12 mm. **D)** Magnetic driving mechanism design of the *Z* (vertical)-axis linear nanopositioner with a working distance of 6 mm.

The open-source *XYZ* nanopositioner (Fig. 2A) has a compact size (21 mm × 21 mm × 40 mm), which is convenient for integration with other systems. Fig. 2B shows the *XYZ* nanopositioner consisting of one vertical and two horizontal linear nanopositioners. As shown in Fig. 2C and 2D, both the horizontal and vertical nanopositioners have a very simple design that utilizes a miniaturized linear slide, neodymium (NdFeB) magnets, a piezo stack actuator, and a connector. The off-the-shelf NdFeB magnets have a chromium coating that provides high surface hardness and corrosion resistance to protect the core of the magnet. The piezo stack actuates the magnet attached to the linear slide and drives the platform to achieve linear positioning. The chromium coating of the magnet also acts as a solid lubricant that reduces the wear on the side wall of the linear slide. Depending on the driving signal waveform (triangular or sawtooth) to the piezo stack, the *XYZ* nanopositioner can provide a range of approximately 3.7 µm in high-resolution scanning mode, a 12 mm long coarse movement in long-range stepping mode, and payload positioning capability up to 12 kg (see video ‘Positioning 12 kg Granite.wmv’ in the file repository).

**Fig. 3:**
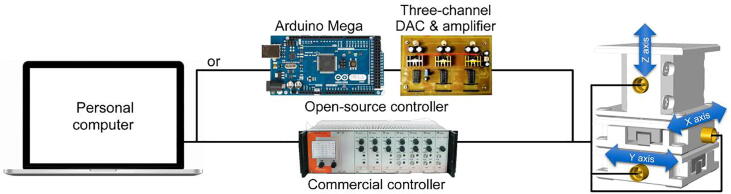
Schematic of controllers for the *XYZ* nanopositioner.

The *XYZ* nanopositioner can be driven by controllers with different voltages (e.g., AttoCube ANC/AMC 300) ranging from − 30 to 150 V for the stepping and scanning modes. Herein we also present a simple, safe, and low-cost open-source controller (Fig. 3) that integrates an Arduino Mega microcontroller, digital-to-analog converters (DACs), and voltage/current amplifiers. The open-source controller can provide a voltage of 0–35 V to drive the nanopositioner for stepping-mode operation.

The *XYZ* nanopositioner was tested for its applicability in various systems, including long-range high-resolution sample positioning inside a scanning electron microscope (SEM), as a micro-structure alignment device for a vibrometer, and in the approaching/scanning mechanism for atomic force microscopy (AFM).

In summary, the presented open-source *XYZ* nanopositioner provides:

• atomic-scale scanning resolution,
• nanometer-scale positioning resolution,
• centimeter-scale working distance,
• heavy load capacity (up to 12 kg in the horizontal positioner),
• simple design and low component cost,
• ease of assembly without the need for adjustments, and
• compact size and ultra-high vacuum compatibility.

## Design files summary

### Key components

The parts were designed using SolidWorks 2014 (Dassault Systèmes SolidWorks Corporation, Waltham, MA, USA) computer-aided design (CAD) software. All the design files are available in IGES format and can be downloaded from the linked Open Science Framework (OSF) file repository. All metal parts were machined with 3000-series aluminum.

**Table:** 

Design file name	File type	Open source license	Location of the file
H-Nano-01.sldprt	CAD	CC BY-SA 4.0	https://doi.org/10.17605/osf.io/7fk3u
H-Nano-02.sldprt	CAD	CC BY-SA 4.0	https://doi.org/10.17605/osf.io/7fk3u
V-Nano-01.sldprt	CAD	CC BY-SA 4.0	https://doi.org/10.17605/osf.io/7fk3u
V-Nano-02.sldprt	CAD	CC BY-SA 4.0	https://doi.org/10.17605/osf.io/7fk3u
V-Nano-03.sldprt	CAD	CC BY-SA 4.0	https://doi.org/10.17605/osf.io/7fk3u
H-Nano-01.slddrw	CAD	CC BY-SA 4.0	https://doi.org/10.17605/osf.io/7fk3u
H-Nano-02.slddrw	CAD	CC BY-SA 4.0	https://doi.org/10.17605/osf.io/7fk3u
V-Nano-01.slddrw	CAD	CC BY-SA 4.0	https://doi.org/10.17605/osf.io/7fk3u
V-Nano-02.slddrw	CAD	CC BY-SA 4.0	https://doi.org/10.17605/osf.io/7fk3u
V-Nano-03.slddrw	CAD	CC BY-SA 4.0	https://doi.org/10.17605/osf.io/7fk3u
Open-Source *XYZ* Nanopositioner assembled.zip	CAD	CC BY-SA 4.0	https://doi.org/10.17605/osf.io/7fk3u
DAC board – Bill of Materials.xlsx	CAD	CC BY-SA 4.0	https://doi.org/10.17605/osf.io/7fk3u
DAC board – PCB layout.pdf	CAD	CC BY-SA 4.0	https://doi.org/10.17605/osf.io/7fk3u
DAC board – Schematic.pdf	CAD	CC BY-SA 4.0	https://doi.org/10.17605/osf.io/7fk3u
DAC board – Gerber files.zip	CAD	CC BY-SA 4.0	https://doi.org/10.17605/osf.io/7fk3u
Arduino Code – Sawtooth generator.ino	Code	CC BY-SA 4.0	https://doi.org/10.17605/osf.io/7fk3u

### 3D-printing files

Files for a 3D-printed version of the *XYZ* nanopositioner are provided in the stereolithography (STL) format and are available on the same OSF file repository. All parts were printed on a fused deposition 3D printer (Prusa i3 MK2.5S, Prusa Research, Prague, Czech Republic) with a 0.4 mm nozzle, 0.1 mm layer height, and 30% infill. The 3D-printed parts were fabricated from polylactic acid (PLA) filaments (Reprap.me, Hedehusene, Denmark).

**Table:** 

Design file name	File type	Open source license	Location of the file
H-Nano-01.STL	CAD	CC BY-SA 4.0	https://doi.org/10.17605/osf.io/7fk3u
H-Nano-02.STL	CAD	CC BY-SA 4.0	https://doi.org/10.17605/osf.io/7fk3u
V-Nano-01.STL	CAD	CC BY-SA 4.0	https://doi.org/10.17605/osf.io/7fk3u
V-Nano-02.STL	CAD	CC BY-SA 4.0	https://doi.org/10.17605/osf.io/7fk3u
V-Nano-03.STL	CAD	CC BY-SA 4.0	https://doi.org/10.17605/osf.io/7fk3u

### Arduino code

The Arduino code generates 8-bit digital signals (through the Arduino Mega ports A, C, and L) to control the DAC board. The predefined stepping frequency is 5 Hz, but this can be changed by modifying the OCR4A value (from 12,500 to 20) in the code. The predefined pull-down control pins (A8–A13) connected to three switches (three-way toggle) can be used to control the movement of the *X*-, *Y*-, and *Z*-axis nanopositioners.

### Electronics

**Fig. 4:**
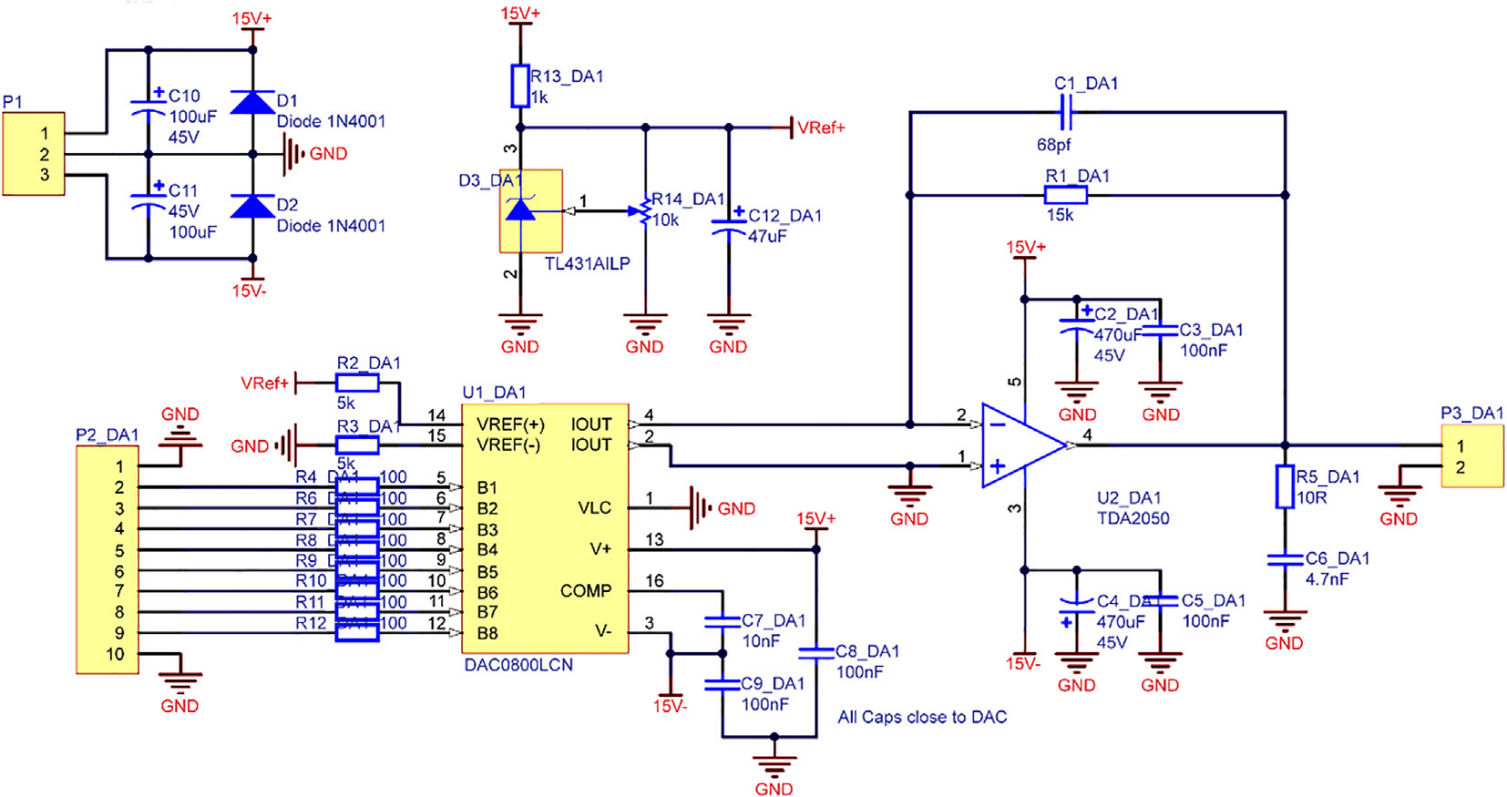
Schematic of the DAC board circuits.

**Fig. 5:**
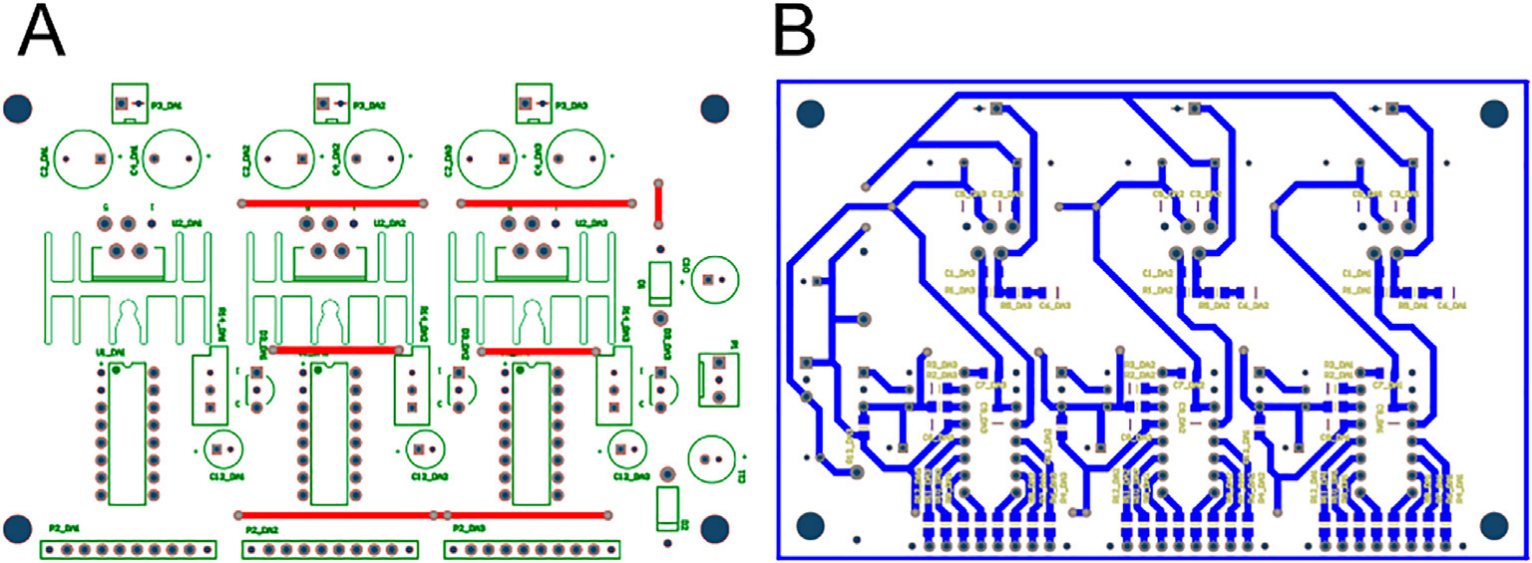
Layout of the DAC printed circuit boards (PCB). The PCB can be fabricated by a PCB manufacturing service or a desktop milling system. To reduce the production cost, instead of using double-sided circuit board, six wires were used on the front side of the PCB. **A**) PCB layout on the front. **B**) PCB layout on the reverse.

The simple, low-cost, and open-source controller comprises an Arduino Mega microcontroller and a DAC board that contains three DACs and current/voltage amplifiers The digital signals generated by the Arduino Mega are fed to the DAC IC which outputs sawtooth waveforms with 8-bit resolution for the long-range stepping mode (Fig. 1.B) Fig. 4 shows a schematic circuit diagram of the DAC board, which requires only one side circuit, resulting in simplicity and cost reduction (Fig. 5). An audio power amplifier IC (TDA2050, STMicroelectronics) amplifies the sawtooth waveform to a maximum voltage of 35 V and peak current of 5 A.

**Fig. 6:**
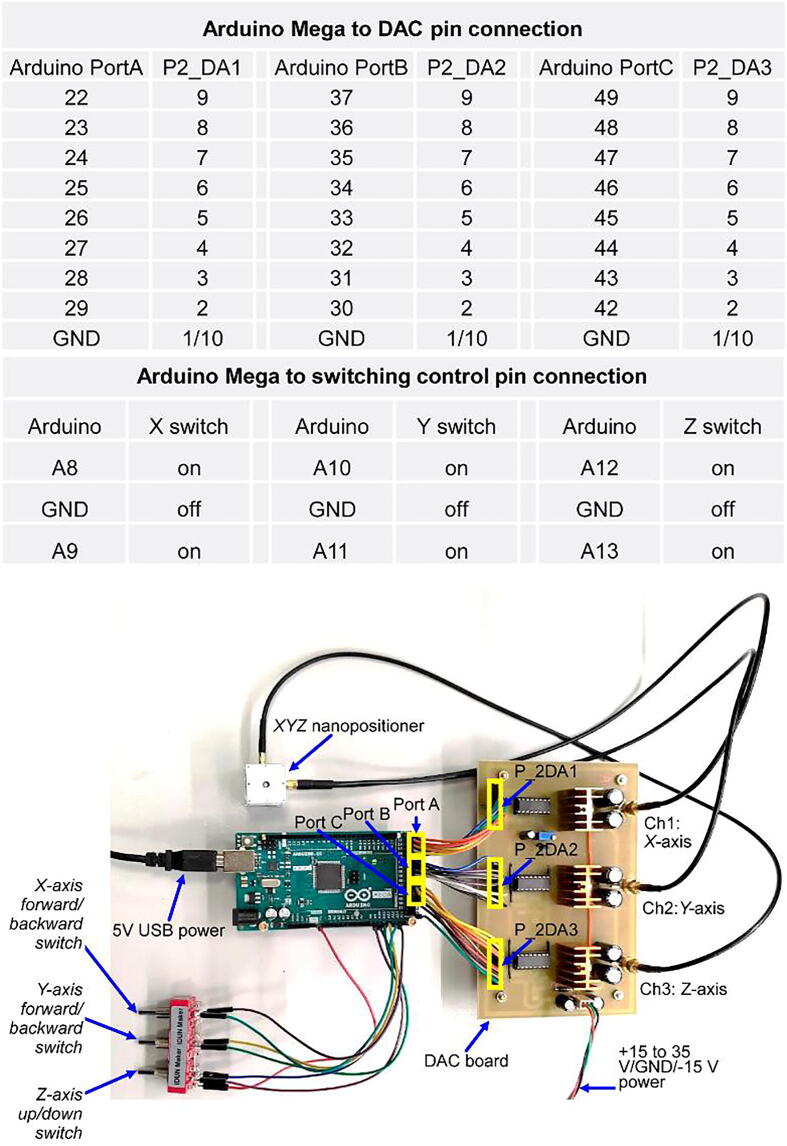
Details of the pin connections between the Arduino Mega controller, DAC board, switches, and the XYZ nanopositioner. Arduino-based open-source controller provides personal computer-independence and easy control for the *XYZ* nanopositioner (see video ‘Arduino controller for *XYZ* Nanopositioner.mp4′ in the photo and video folder of the file repository).

The DAC board provides three channels for driving the *XYZ* nanopositioner for long-range stepping-mode applications. Gerber and Drill files are also available in the OSF file repository. The *XYZ* nanopositioner can be driven by a p-p sawtooth signal of 15 V, and the user can change the power voltage of the DAC board from +15 to +35 V to obtain a p-p driving signal of 35 V. Fig. 6 details the pin connections between the Arduino Mega controller, DAC board, switches, and the *XYZ* nanopositioner.

## Bill of materials summary

Apart from the 3D-printed components, some items were purchased from other sources. In addition, the metal parts were prepared by a local machine shop.

**Table:** 

Designator	Component	Number	Cost per unit – EUR	Total cost – EUR	Source of materials	Material type
H-Nano-01	Horizontal nanopositioner part 01	2	72	144	Machine shop	Metal
H-Nano-02	Horizontal nanopositioner part 02	2	34	68	Machine shop	Metal
V-Nano-01	Vertical nanopositioner part 01	1	86	86	Machine shop	Metal
V-Nano-02	Vertical nanopositioner part 02	1	54	54	Machine shop	Metal
V-Nano-03	Vertical nanopositioner part 03	1	42	42	Machine shop	Metal
Connector	MMCX jack, female Socket	3	2.1	6.3	Digi-key	Metal
Piezo stack	Piezo actuator type-F (AE0203D04)	3	22.5	67.5	MMech	Ceramic
Linear slide 8–21	Nippon Bearing miniature slide table SYBS 8–21	2	58.3	116.6	Nippon Bearing	Metal
Linear slide 6–13	Nippon Bearing miniature slide table SYBS 6–13	1	62.49	62.49	Nippon Bearing	Metal
M1 screw	FPHM1-0.25x2.2 (2.5,0.3) CR3/B1	2	0.03	0.06	Hamanaka Shoukin	Metal
M1.2 screw	FPHM1.2–0.25x1.5 (2.8, 0.2) Ni	2	0.03	0.06	Hamanaka Shoukin	Metal
M1.4 screw	FPHM1.4–0.3x3 (2.5, 0.3) Ni coating	12	0.03	0.36	Hamanaka Shoukin	Metal
M2 screw	FPHM2-0.4x2.5 (1.5, 0.8) Ni coating	4	0.03	0.12	Hamanaka Shoukin	Metal
Magnet	3 mm magnetic cube (NdFeB Magnets 03*03)	6	0.18	1.08	TopMagnet	Metal
Cable	SMA (M) – MMCX (M) cable (CBA-SMAMR-MMCXM-ND)	3	5.9	17.65	Digi-key	Composite
Arduino	Arduino Due board	1	35.8	35.8	Digi-key	Semiconductor
DAC board	See DAC board – Bill of Materials.xlsx	1	63.62	63.62	See bill of materials	Semiconductor

## Build instructions

The assembly requires instant glue (Loctite Super Liquid 20 GR, Henkel, Düsseldorf, Germany) to ensure firm bonding between the components. This instruction can be applied to both metal and 3D-printed parts in the *XYZ* nanopositioners.

**Fig. 7:**
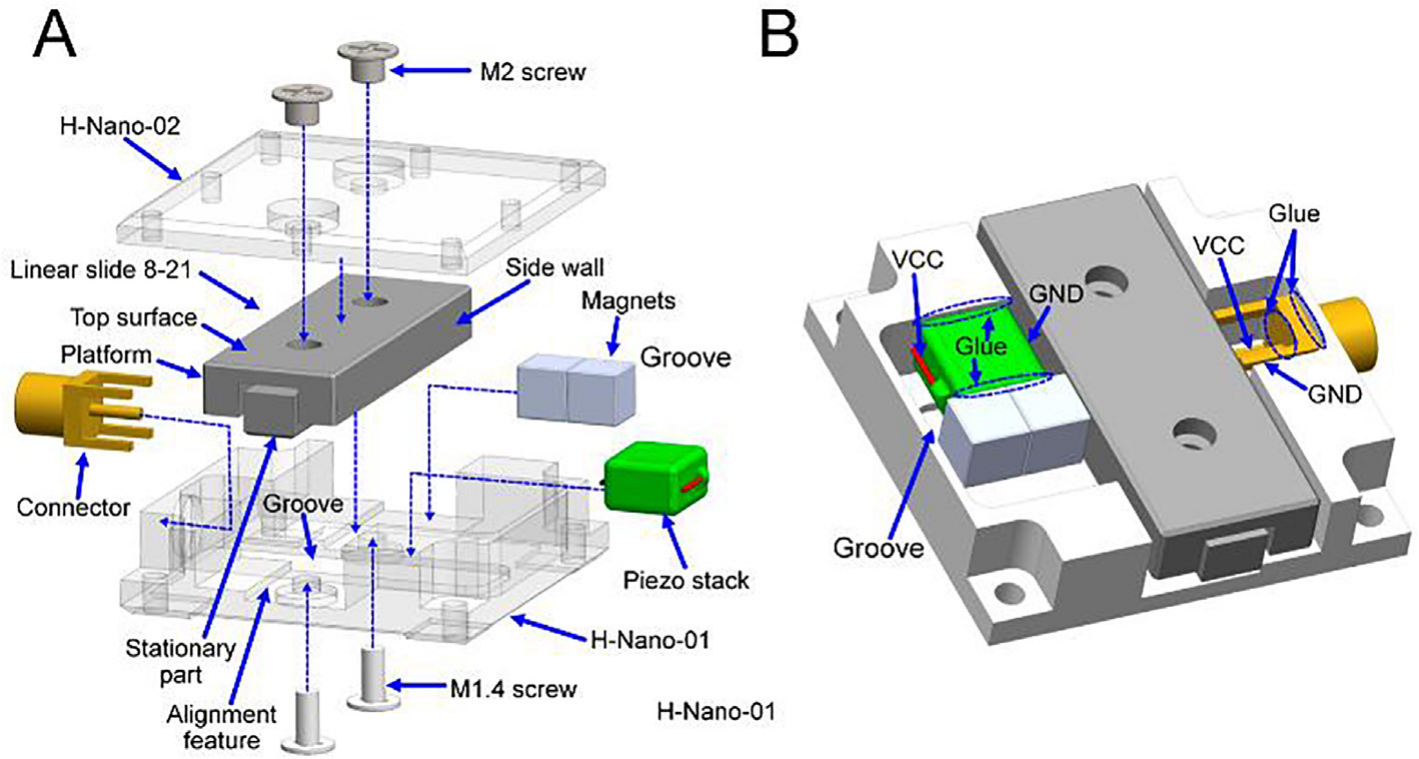
Horizontal nanopositioner assembly: **A)** assembly process and **B)** gluing points and electrical connections.

Horizontal nanopositioner assembly (Fig. 7)

1. Components needed: 4 × M2 screws, 4 × M1.4 screws, 4 × magnets, 2 × piezo stack, 2 × connector, 2 × linear slide 8–21, 2 × H-Nano-01, and 2 × H-Nano-02.
2. Insert and glue the connector to the side hole of the H-Nano-01.
3. Solder the voltage common collector (VCC) and ground (GND) wires (placed inside a groove on H-Nano-01) of the piezo stack to the connector. Apply glue to the VCC pin of the connector (Fig. 7B).
4. Place the stationary part of the linear slide in contact with the alignment feature (Fig. 7A) of H-Nano-01.
5. Fix the linear slide on the H-Nano-01 with two M1.4 screws.
6. Glue the piezo stack on the H-Nano-01.
7. Attach two magnets to the sidewall of the linear slide platform. Ensure that the magnets are >0.1 mm below the platform top surface.
8. Glue the magnets to the piezo stack.
9. Fix the H-Nano-02 to the platform with two M2 screws.
10. Repeat the above steps for the second horizontal positioner assembly.
11. For a detailed sequence of the assembly, see video ‘Assembly Animation H Nano.mp4′ in the file repository.

**Fig. 8:**
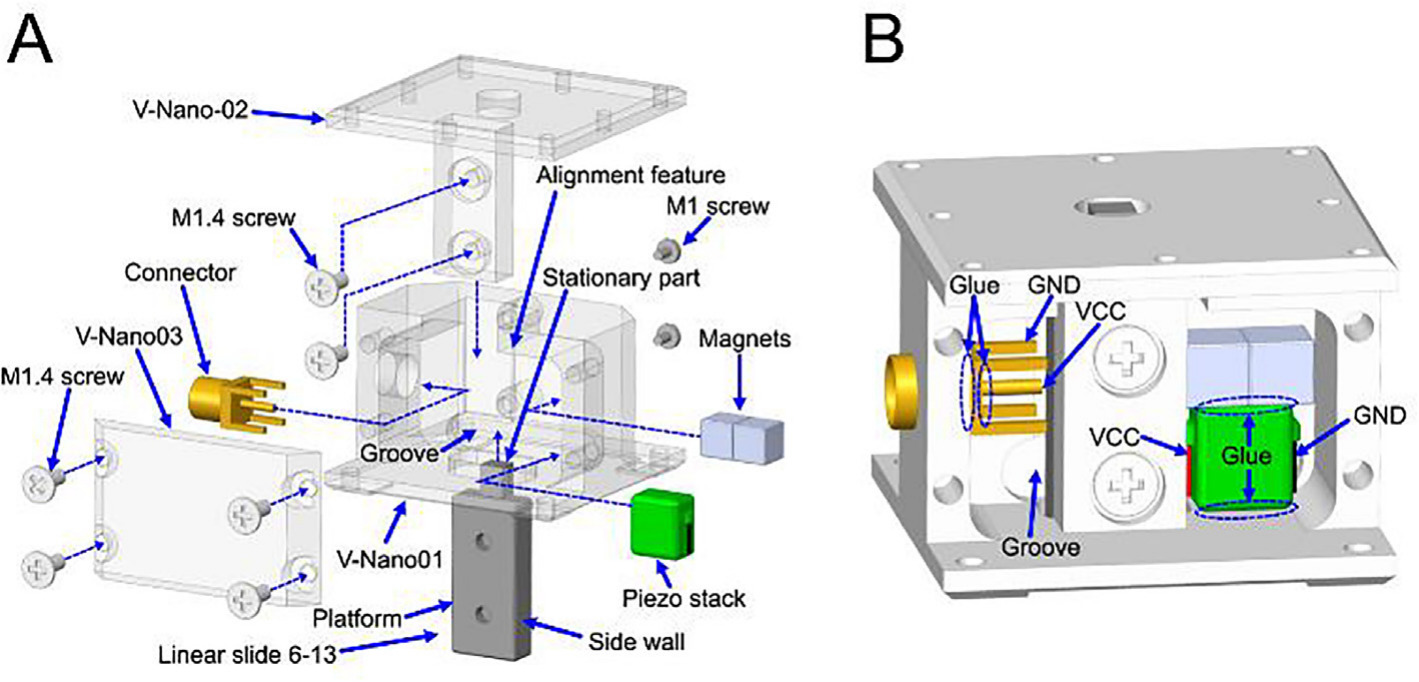
Vertical nanopositioner assembly: **A)** assembly process and **B)** gluing points and electrical connection.

Vertical nanopositioner assembly (Fig. 8)

1. Components needed: 2 × M1 screws, 6 × M1.4 screws, 2 × magnets, 1 × piezo stack, 1 × connector, 1 × linear slide 6–13, 1 × V-Nano-01, 1 × V-Nano-02, and 1 × V-Nano-03.
2. Insert and glue the connector to the side hole of the V-Nano-01.
3. Solder the VCC and GND wires (placed inside a groove on the V-Nano-01) of the piezo stack to the connector. Apply glue to the VCC pin of the connector (Fig. 8B).
4. Place the stationary part of the linear slide in contact with the alignment feature (Fig. 8A).
5. Fix the linear slide on the V-Nano-01 with two M1 screws.
6. Glue the piezo stack on the V-Nano-01.
7. Stick two magnets on the side wall of the linear slide.
8. Glue the magnets to the piezo stack.
9. Fix the V-Nano-02 to the platform of the linear slide using two M1.4 screws.
10. Fix the V-Nano-03 to the V-Nano-01 with four M1.4 screws.
11. For a detailed sequence of the assembly, see video ‘Assembly Animation V Nano.mp4′ in the file repository.

## Operating instructions

1. Connect the Arduino Mega to a PC with a universal serial bus (USB) cable and load the sawtooth generator code.
2. Connect the DAC board and the switches to the Arduino Mega.
3. Connect the + 15–35 V supply, ground, and − 15 V supply to the DAC board.
4. Connect the *XYZ* nanopositioner to the three SMA connectors on the DAC board.
5. Adjust the variable resistor on the DAC board to maximize the sawtooth signal output.
6. Use the *X* ,*Y*, and *Z* switches to control *X* ,*Y*, and *Z* directions, respectively.
7. See video ‘Arduino controller for *XYZ* Nanopositioner.mp4′ in the OSF file repository.

## Validation and characterization (metal-based *XYZ* nanopositioner)

To characterize the performance of the *XYZ* nanopositioner, the stabilities of both high-resolution scanning and long-range stepping modes (driven by an ANC 300 controller, AttoCube, Haar, Germany) were tested using a laser interferometer (SP-S series, SIOS Meßtechnik GmbH, Ilmenau, Germany). Moreover, resonant frequencies of the mechanism were measured to examine the mechanical stiffness and dynamic response to environmental vibrations.

### High-resolution scanning mode characterization

**Fig. 9:**
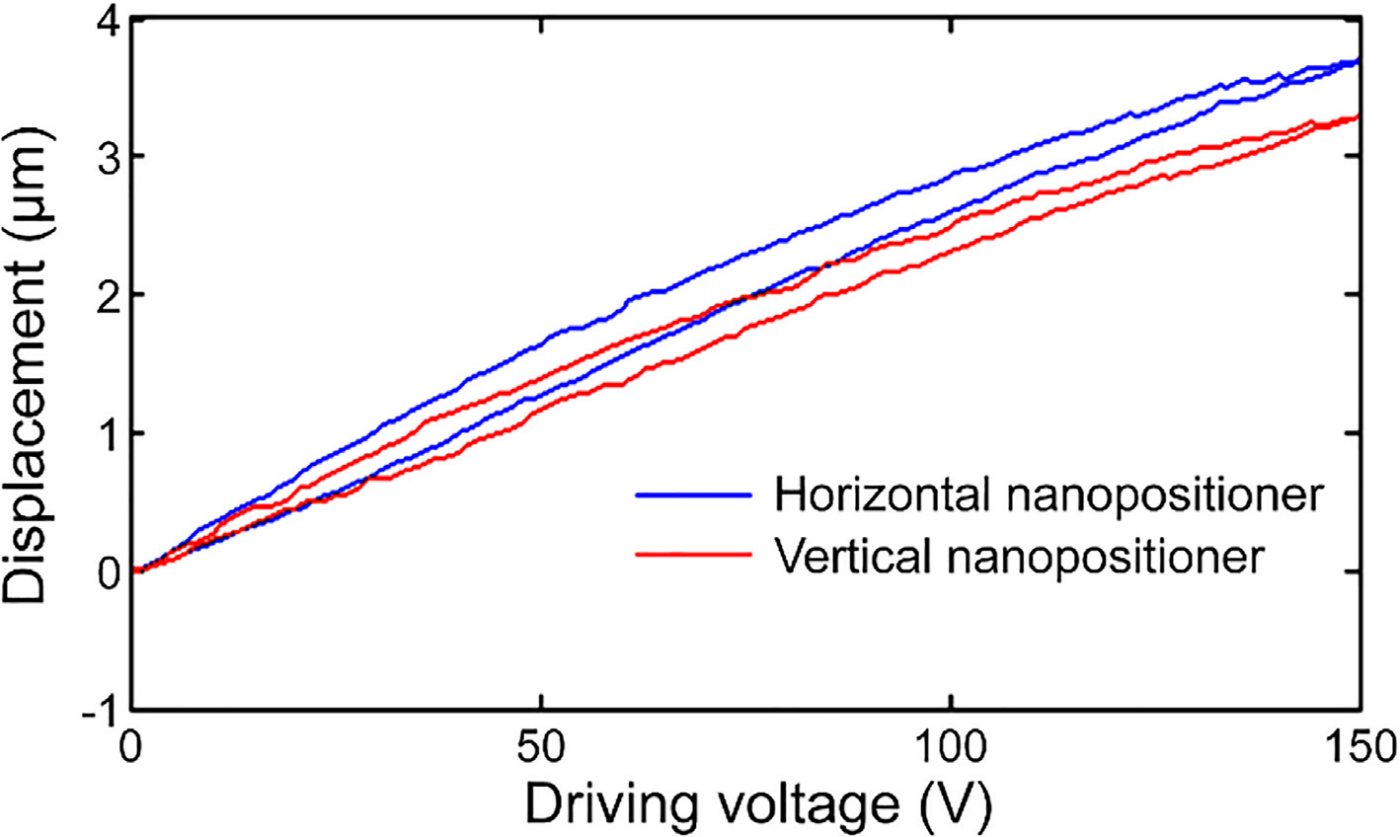
Scanning displacements of the horizontal and vertical nanopositioners in high-resolution scanning mode.

In the high-resolution scanning mode tests, the displacements of the horizontal and vertical nanopositioners were measured separately. The blue and red lines shown in Fig. 9 represent the displacements of the horizontal and vertical nanopositioners, respectively, driven by a triangular waveform with a voltage in the range 0–150 V. The results show that the full-scan ranges of the horizontal and vertical nanopositioners were 3.75 and 3.29 µm, respectively. The short travel range in the high-resolution scanning mode is suitable for high precision applications such as high-resolution imaging in AFM [29]. Moreover, the average slopes of the displacement versus driving voltage curves were approximately 25.0 nm/V and 21.9 nm/V for the horizontal and vertical nanopositioners, respectively. The slopes can be used to convert the electrical noise of the driving controller into displacements for evaluating the spatial resolution. By using a commercial AttoCube controller with an electrical noise of 5 mV (peak to peak, bandwidth = 20 MHz), sub-nanometer precision (horizontal: 0.125 nm, vertical: 0.11 nm) can be achieved.

### Long-range stepping mode characterization

**Fig. 10:**
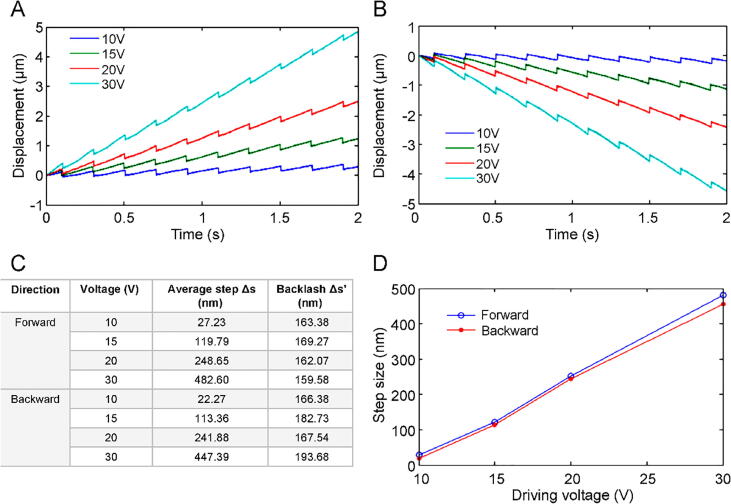
Stepping displacement of the horizontal nanopositioner in **A)** forward and **B)** backward directions. **C)** Driving voltage and average step size. **D)** Comparison of forward and backward directions.

**Fig. 11:**
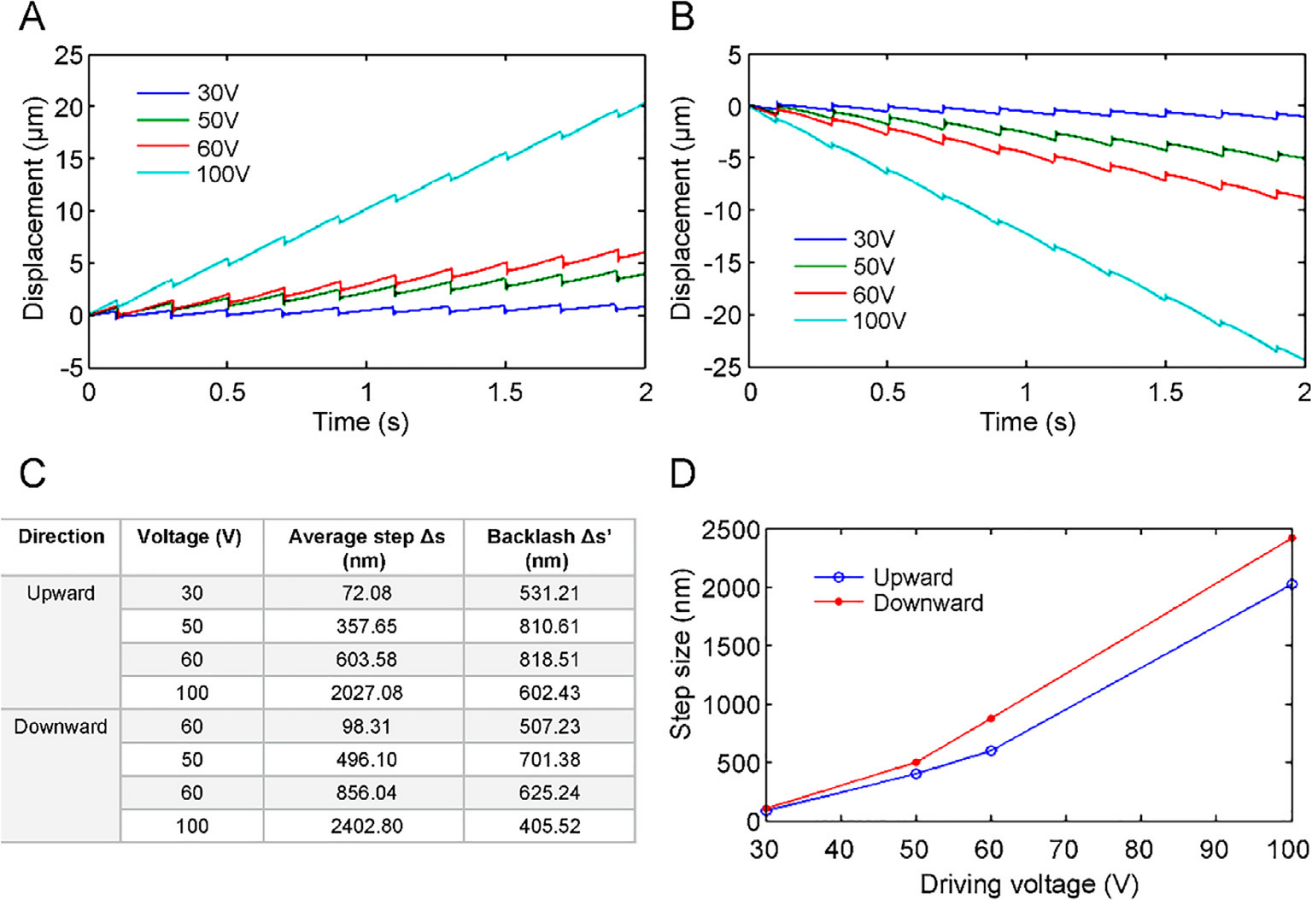
Stepping displacement of the vertical nanopositioner in **A)** upward and **B)** downward directions. **C)** Driving voltage and average step size. **D)** Comparison between upward and downward directions.

A sawtooth waveform with a frequency of 5 Hz was utilized to drive the nanopositioners in the long-range stepping mode. Fig. 10A and B show the forward and backward displacements, respectively, of the horizontal nanopositioner at a driving voltage range from 10 to 30 V. Fig. 10C and D shows that the average step size is proportional to the driving voltage, and the step sizes in the forward and backward directions are similar. The stepping displacements of the vertical nanopositioner in the upward and downward directions are shown in Fig. 11A and B, respectively. The average step size is positively proportional to the driving voltage between 30 and 100 V (Fig. 11C and D). The upward step size is smaller than the downward step size owing to the effect of gravity. In addition, the horizontal nanopositioner can carry a load of 12 kg, which is the maximum load capacity of the linear slide. The vertical nanopositioner load capacity was limited to 50 g because of the limitations presented by the friction force between the magnet and the sidewall of the linear slide. These results confirm that the proposed nanopositioner can achieve a step resolution of tens of nanometers, which is competitive with commercial stepper positioners widely used in nanoscale manipulation [30], [31], [32].

### Resonant frequency characterization

**Fig. 12:**
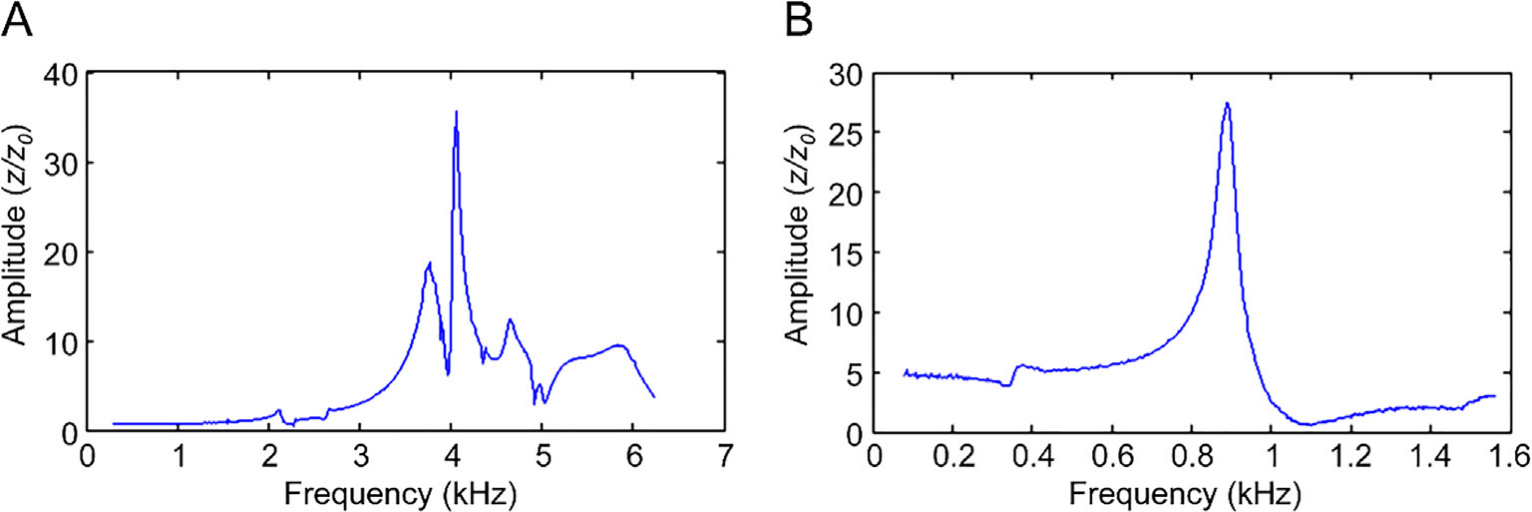
Resonant spectra of the **A)** horizontal nanopositioner (single axis) and **B)***XYZ* nanopositioner (three axes).

In the resonance tests, the horizontal nanopositioner was tuned using a sinusoidal waveform, and the vibrations in the vertical direction were measured using a laser interferometer. The system frequency response was obtained using a lock-in amplifier (SR830, Stanford Research Systems). The resonant spectrum of the horizontal nanopositioner (Fig. 12A) shows that the main resonant peak is located at 4.06 kHz. While testing the *XYZ*-stacked nanopositioner, the vertical nanopositioner was driven, and the vertical vibrations were measured. The result (Fig. 12B) shows that the resonant frequency of the *XYZ*-stacked nanopositioner is 0.89 kHz. The resonant frequency is an index for examining the speed and rigidity of the nanopositioner [19] and the resonant frequencies of the proposed nanopositioners are adequate for general applications [33], [34], [35].

## Demonstrations and applications

The proposed nanopositioner has been utilized in multiple scientific research applications. Practical tests and demonstrations were carried out, wherein the *XYZ* nanopositioner was integrated into a SEM, vibrometer, and an AFM.

### Sample probing inside a SEM

**Fig. 13:**
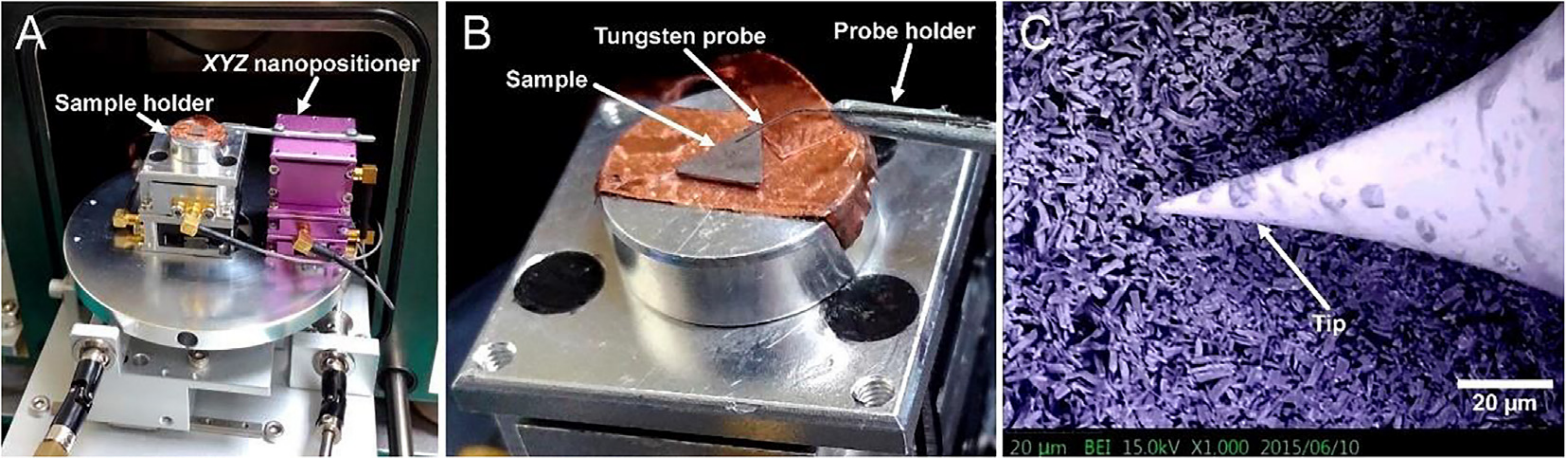
*XYZ* nanopositioner utilized inside a table-top SEM. **A)** Photograph of the *XYZ* nanopositioner beside the *X–Y* positioner of the SEM on a sample stage. **B)** Photograph of the tungsten probe attached to the probe holder showing the sample under the probe before imaging. **C)** Image of the SEM tip (end radius <10 nm) at the end of the tungsten probe used to measure the sample surface.

The *XYZ* nanopositioner is compact, which enables easy integration on a sample stage inside a desktop SEM (EM100, TEMIC, Taipei, Taiwan). The *XYZ* nanopositioner, driven by the Arduino-based open-source stepping mode controller, was used to achieve the high-resolution nanoscale probing of a ZnO nanorod surface, as shown in Fig. 13A. The nanopositioner was fitted with a probe holder having an attached tungsten probe (Fig. 13B). During operation, the tip of the tungsten probe was actuated in three different axes on top of the ZnO nanorod sample for electrical characterization, as shown in Fig. 13C. The nanopositioner could be operated in a 10^−4^ Torr vacuum.

The platform of the linear slide was made of ferromagnetic material and magnets were attached to the platform. Therefore, half the magnetic field travelled within the platform and the other half was partially shielded by the metal components. Moreover, the distance between the magnets inside the nanopositioner and the target objects was greater than 25 mm. The magnetic field of the nanopositioner decreases considerably with distance (I = 1/d^2^, where *d* is the distance and *I* is the intensity of the magnetic field); hence, there were no observable distortions during the SEM imaging. Thus, the magnet-based driving mechanism did not affect the SEM imaging quality when the magnets were away from the sample.

### Microstring resonator characterization

**Fig. 14:**
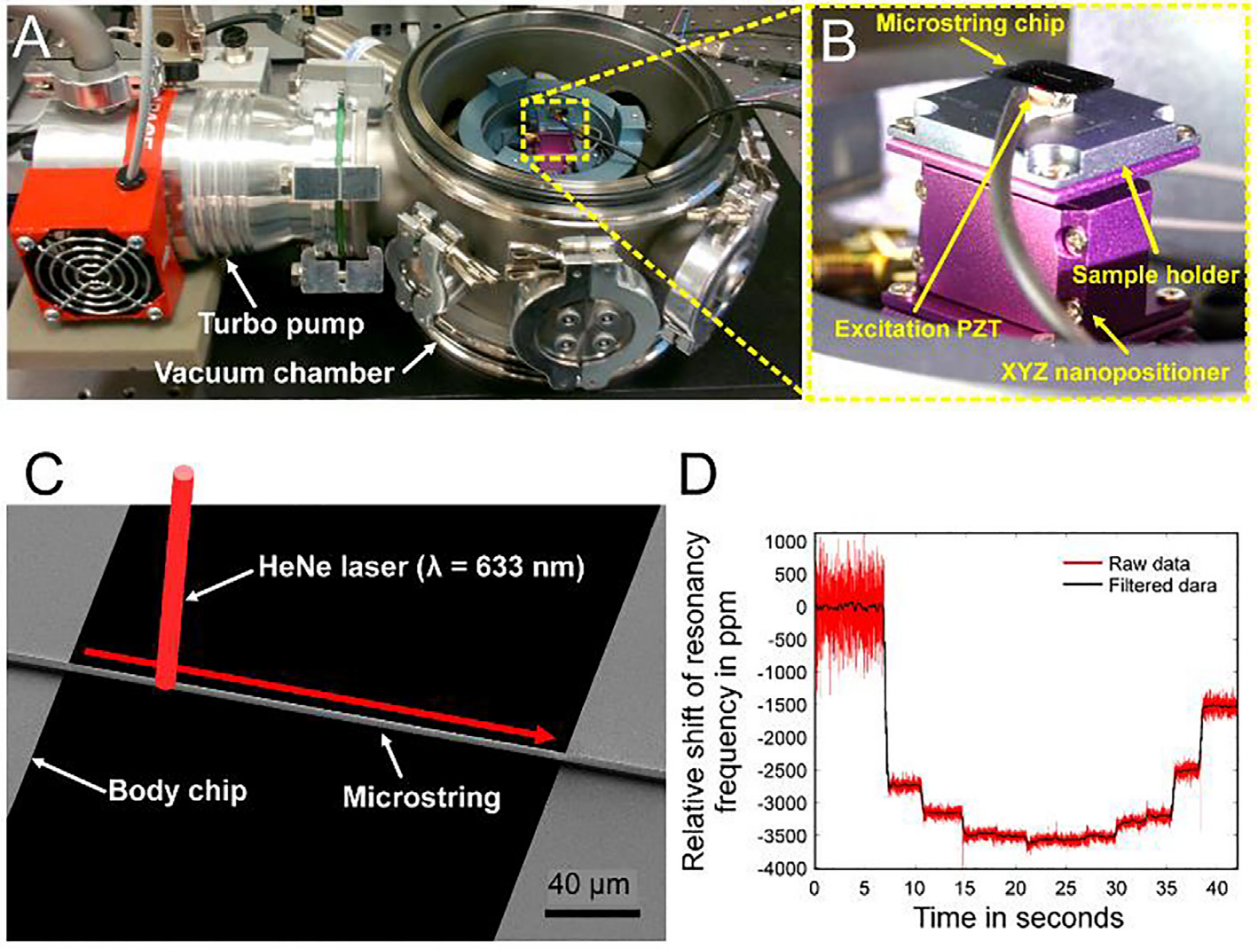
*XYZ* nanopositioner integrated in a custom vibrometer. **A)** Photograph of the *XYZ* nanopositioner inside a vacuum chamber. **B)** Photograph of a microstring chip and an excitation PZT fixed on a sample holder on top of the nanopositioner. **C)** Image showing the direction of the HeNe laser scanned along the microstring used to measure the change in the resonance frequency simultaneously. **D)** Plot of the resonance frequency of the microstring, which shifted as the laser was focused on different parts of the microstring.

As shown in Fig. 14A, the *XYZ* nanopositioner was placed inside a small vacuum chamber (<10^−3^ Torr) for ultra-sensitive microstring resonator characterization [36]. A cleanroom-fabricated silicon chip with suspended SiN microstrings was placed on top of a piezoelectric actuator (PZT) for actuation (Fig. 14B). A laser Doppler vibrometer (MSA-500, Polytec, Baden-Württemberg, Germany) was used to measure the vibrational displacement, and a lock-in amplifier (HF2LI 50 MHz, Zurich Instruments) was employed to track the heat-induced resonance frequency changes. The nanopositioner played a crucial role in precisely aligning specific microstrings within the optical field of view of the vibrometer. As shown in Fig. 14C, the vibrometer laser (633 nm HeNe) was scanned along the length of the microstring (200 µm × 3 µm × 0.2 μm), and the fundamental resonance frequency was tracked. The decreasing thermal conductance towards the center of the microstring leads to an increase in the average temperature, and the resulting thermal expansion was measured by detuning the resonance frequency, as shown in Fig. 14D. The nanopositioner has also been applied in a digital video disc (DVD) optical pick-up unit (OPU) sensing system [37], [38], [39], [40], [41], [42], [43], [44], [45]-based vibrometer, as reported previously [46].

### Atomic resolution imaging

**Fig. 15:**
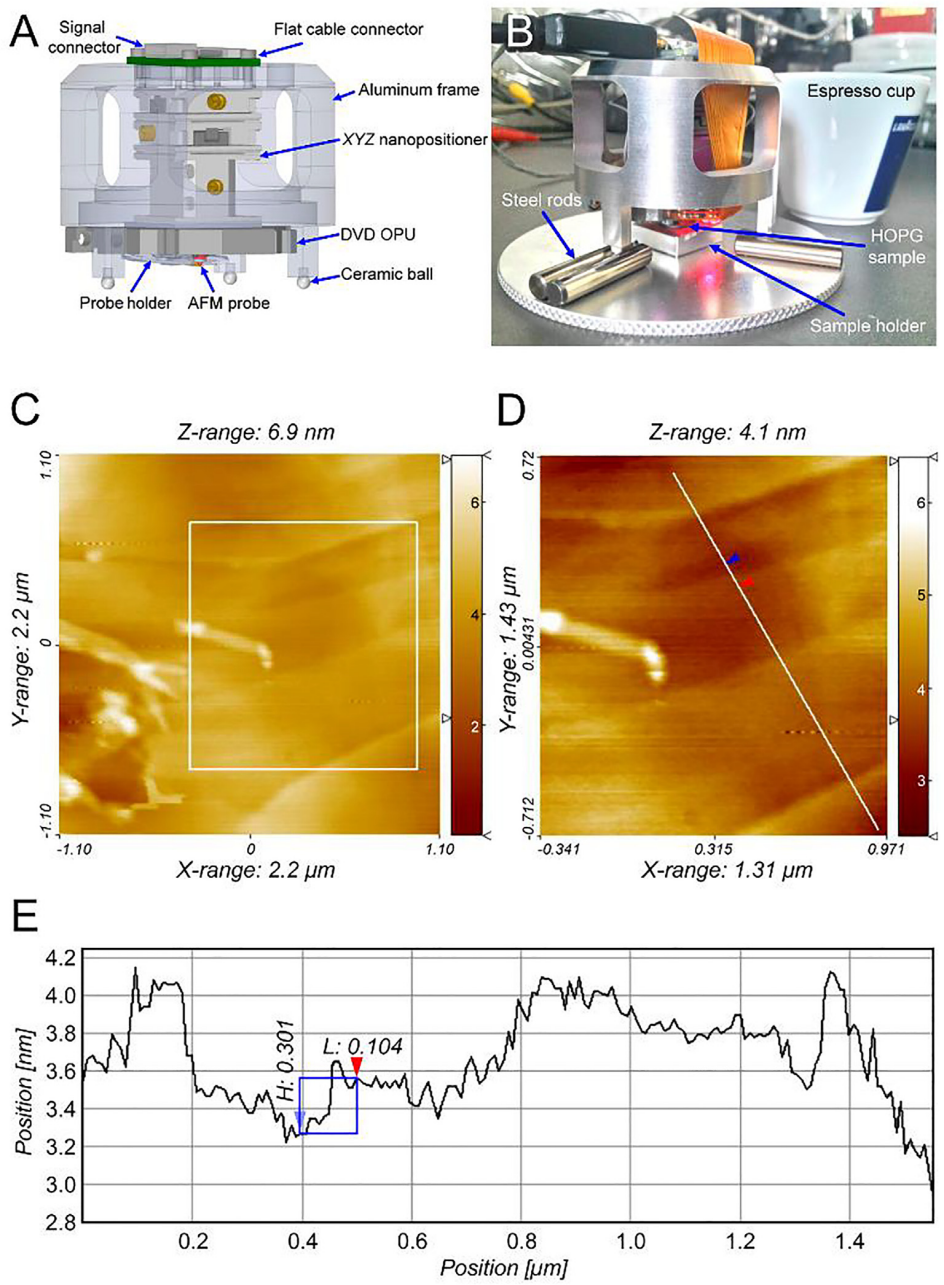
An *XYZ* nanopositioner-based miniaturized 'Espresso AFM.' **A)** Schematic of the ‘Espresso AFM’ (diameter: 6 cm, height: 5 cm). **B)** Photograph of the 'Espresso AFM.' The AFM was placed on top of six steel rods by a kinematic mounting method. A HOPG sample was placed under the 'Espresso AFM.' **C)** Topography of the HOPG sample surface **D)** Magnified image of the HOPG surface. **E)** Cross sectional analysis of the measurement result. A single-carbon-atom step (0.3 nm) on the HOPG surface is indicated by the blue and red arrows. (For interpretation of the references to colour in this figure legend, the reader is referred to the web version of this article.)

For AFM instrumentation, the *XYZ* nanopositioner provides an elegant solution to miniaturize the system size. In particular, reducing the size of the device enhances the rigidity of the interaction between the AFM probe and the sample. Fig. 15A shows a schematic of a miniaturized AFM—denoted ‘Espresso AFM’ owing to a size similar to that of an espresso cup (Fig. 15B)—utilizing the nanopositioner and a DVD OPU for nanoscale imaging [47], [48], [49], [50], [51], [52], [53]. The nanopositioner provides long-range stepping and high-resolution scanning modes, which are convenient for *X*–*Y* axes coarse adjustment/*Z*-axis tip motion and atomic-resolution imaging, respectively. Crucially, the DVD OPU monitors the AFM probe at atomic resolution [54], [55], [56], [57], [58], [59], [60]. To characterize the performance of the ‘Espresso AFM’ system, a highly oriented pyrolytic graphite (HOPG) sample was used. Working videos of the ‘Espresso AFM’ can be found in ‘photo and video’ folder of the file repository.

The nanopositioner was used to move the AFM probe toward the HOPG surface, and the surface was then scanned in an area of 2.2×2.2 μm^2^, clearly revealing the graphene layers, as shown in Fig. 15C. The results of the cross-sectional analysis of the line shown in the enlarged image in Fig. 15D is shown in Fig. 15E. As indicated by the two arrows, a single-carbon-atom step (0.3 nm) was imaged by the miniaturized AFM. Furthermore, the high stiffness of the nanopositioner and compact size of the AFM system reduced the mechanical instability to the sub-atomic scale (<0.2 nm).

In conclusion, the open-source *XYZ* nanopositioner achieves:

• easy integration to existing systems,
• nanoscale probing inside an SEM,
• coarse and fine scanning, and
• atomic resolution imaging.

## Design variations

**Fig. 16:**
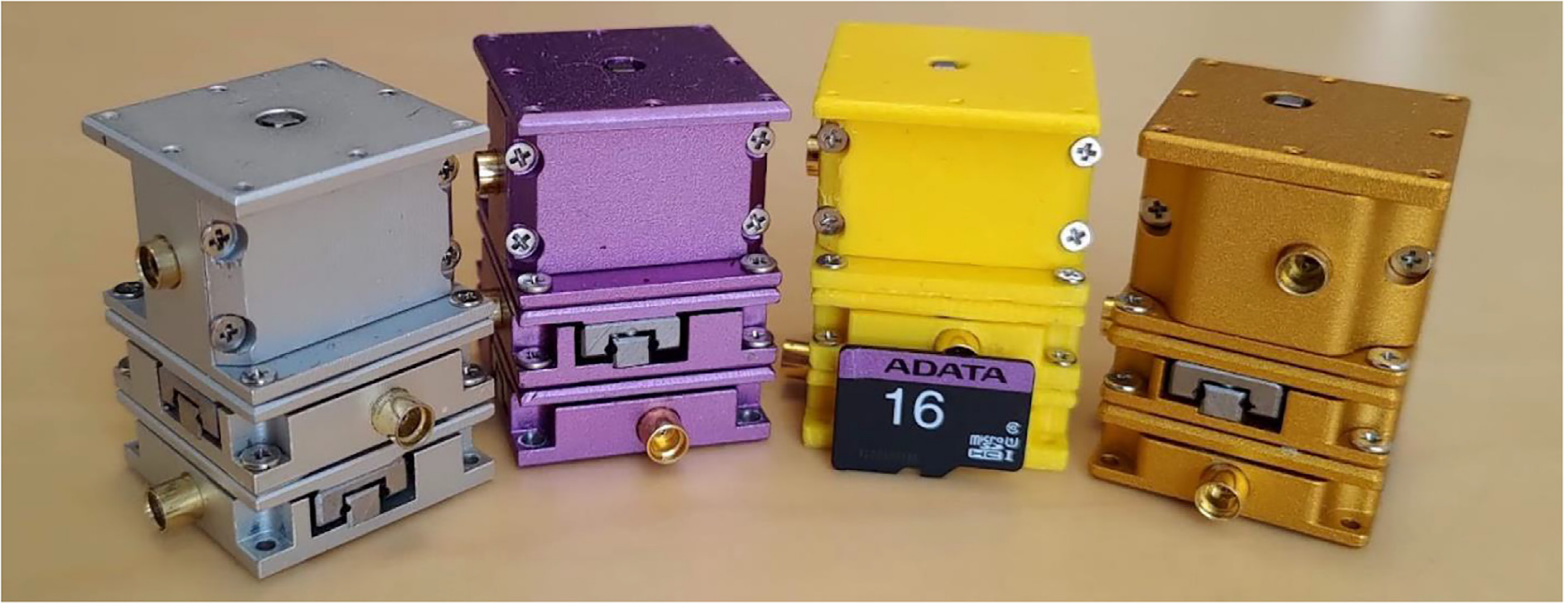
Variations of the *XYZ* nanopositioner: silver and purple nanopositioners made of aluminum parts, a yellow nanopositioner made from 3D-printed parts, and a gold-colored nanopositioner, which is a refined version for easier metal machining. (For interpretation of the references to colour in this figure legend, the reader is referred to the web version of this article.)

The nanopositioner variants (Fig. 16) share the same driving mechanism. In the following list, we provide the CAD images and photographs of the variations for diverse applications: 3D-printable nanopositioners, miniaturized versions, one with a central aperture of 20 mm, hacked conventional cross-roller linear slides, and variations compatible with commercial systems. Working videos of those nanopositioners can be found in ‘photo and video’ folder of the file repository.

**Table:** 

Name	Features	CAD image	Photo	Axis	Working range	Applications
Variation-01	3D-printable *XYZ* nanopositioner			3	X & Y: 12 mm Z: 6 mm	General purpose
Variation-02	Refined *XYZ* nanopositioner			3	X & Y: 12 mm Z: 6 mm	General purpose
Variation-03	3D-printable, double linear slides			1	28 mm	General purpose
Variation-04	3D-printable, AttoCube actuator compatible			1	6 mm	General purpose [61], [62]
Variation-05	AttoCube actuator compatible, low temperature.			1	12 mm	Ultra-high vacuum environment & general purpose
Variation-06	20-mm diameter central aperture			3	X & Y: 12 mm Z: 6 mm	Optical alignment & general purpose
Variation-07	Hacked MISUMI SSEBWM14 linear slide, unlimited range.			1	250 mm	Long range nanopositioning
Variation-08	Hacked Nippon Bearing SYT1025 linear slide			1	12 mm	General purpose
Variation-09	Hacked Nippon Bearing SVTS6360 linear slide			1	235 mm	DNA sample array spotting
Variation-10	Miniature size			2	X & Y: 6 mm	General purpose[63], [64]
Variation-11	21-axis nanoscale probing			21	X & Y: 28 mm Z: 12 mm	High-resolution probing

## Declaration of Competing Interest

The authors declare that they have no known competing financial interests or personal relationships that could have appeared to influence the work reported in this paper.
